# Confidence and quality in managing CKD compared with other cardiovascular diseases and diabetes mellitus: a linked study of questionnaire and routine primary care data

**DOI:** 10.1186/1471-2296-12-83

**Published:** 2011-08-05

**Authors:** Mohammad A Tahir, Olga Dmitrieva, Simon de Lusignan, Jeremy van Vlymen, Tom Chan, Ramez Golmohamad, Kevin Harris, Charles Tomson, Nicola Thomas, Hugh Gallagher

**Affiliations:** 1 Primary Care Informatics, Division of Public Health Sciences and Education, St George's - University of London, Cranmer Terrace, London SW17 0RE, UK; 2 Department of Health Care Management and Policy, University of Surrey, Guildford, UK, Surrey GU2 7XH; 3 University Hospitals of Leicester NHS Trust, John Walls Renal Unit, Leicester General Hospital, Leicester LE5 4PW UK, UK; 4 Department of Renal Medicine, Southmead Hospital, Bristol BS10 5NB, UK; 5 School of Community and Health Sciences, City University London, 20 Bartholomew Close, London EC1A 7QN, UK; 6 SW Thames Renal & Transplantation Unit, St Helier Hospital, Wrythe Lane Carshalton, Surrey SM5 1AA UK, UK

**Keywords:** Renal Insufficiency, Chronic, Primary Care, Blood pressure, Family Practice, Quality of Healthcare, Proteinuria, Medical Records systems, computerised, Reimbursement, incentives

## Abstract

**Background:**

Much of chronic disease is managed in primary care and chronic kidney disease (CKD) is a recent addition. We are conducting a cluster randomised study of quality improvement interventions in CKD (QICKD) - Clinical Trials Registration: ISRCTN56023731. CKD registers have a lower than expected prevalence and an initial focus group study suggested variable levels of confidence in managing CKD.

Our objective is to compare practitioner confidence and achievement of quality indicators for CKD with hypertension and diabetes.

**Method:**

We validated a new questionnaire to test confidence. We compared confidence with achievement of pay-for-performance indicators (P4P) and implementation of evidence-based guidance. We achieved a 74% (148/201) response rate.

**Results:**

87% (n = 128) of respondents are confident in managing hypertension (HT) compared with 59% (n = 87) in managing HT in CKD (HT+CKD); and with 61% (n = 90) in HT, CKD and diabetes (CKD+HT+DM).

85.2% (P4P) and 62.5% (National targets) of patients with hypertension are at target; in patients with HT and CKD 65.1% and 53.3%; in patients with HT, CKD and DM 67.8% and 29.6%.

Confidence in managing proteinuria in CKD is low (42%, n = 62). 87% of respondents knew BP treatment thresholds in CKD, but only 53% when proteinuria is factored in. Male GPs, younger (< 35 yrs), and older (> 54 yrs) clinicians are more confident than females and 35 to 54 year olds in managing CKD.

84% of patients with hypertension treated with angiotensin modulating drugs achieve achieved P4P targets compared to 67% of patients with CKD.

**Conclusions:**

Practitioners are less likely to achieve management targets where their confidence is low.

## Background

The management of cardiovascular disease including hypertension and diabetes is well established in primary care [1-3] and chronic kidney disease (CKD) is a relatively recent addition [4,5]. Computerisation of primary care records and explicit national guidance supported by pay for performance (P4P) targets have resulted in a rapid improvement in chronic disease management in primary care [6-9]. In CKD, guidance prioritises the control of systolic blood pressure in patients with proteinuria and or diabetes and recommends the use of angiotensin modulating drugs for achieving control [10-12].

CKD is a new priority for primary care and little was known about how to improve quality in CKD [13]. Patients with CKD can be readily identified using a simple formula to estimate glomerular filtration rate (eGFR); a diagnosis is generally made when eGFR is under 60 ml/min/1.73 m2 on at least two occasions three months apart. People with CKD have a higher mortality and morbidity principally due to cardiovascular disease [4]. Hypertension and diabetes were included in P4P since its inception in 2004. CKD was added in 2006 with performance indicators for: creating a disease register, measuring and controlling BP and proteinuria, and treating high risk patients with angiotensin converting enzyme inhibitors [14]. However, the prevalence of CKD reported when it was introduced as an indicator in 2006 was only half of that expected from epidemiological studies, suggesting under-ascertainment [15]. We carried out a diagnostic analysis to explore the factors limiting the achievement of quality improvement [16]. This showed that primary care professionals had gaps in their knowledge, highly variable views about this condition and lacked confidence in explaining and managing the condition [17].

We carried out this questionnaire study to assess primary care clinician confidence in the management of CKD and to explore whether confidence may be associated with the quality of care.

## Methods

### Subjects

The subjects of this research are practitioners working in 30 practices drawn from the 127 practices in the Quality Improvement in Chronic Kidney Disease trial (QICKD Clinical Trials Registration: ISRCTN56023731).

At the time of this study we were conducting a cluster randomised trial (CRT) of quality improvement interventions in CKD [18] which includes conducting a process evaluation to explore the reasons why the trial may, or may not succeed [19]. The CRT has three arms: "Audit based education" (feedback of performance compared with peers in an educational context) [20]; "Guidelines and prompts" (postal reminders about management of CKD and copies of NICE guidance [15]); and usual practice. The study has 25 practices per arm to provide sufficient power to detect a change in systolic BP, our primary outcome measure, between the arms. The process evaluation involves six practices which participate in an in-depth evaluation involving focus groups, and an additional 10 practices per arm which form a "Questionnaire group" in order to explore the impact of the interventions on confidence in the management of CKD. The evaluation practices are excluded from the final analysis of the CRT to avoid the possibility of contamination.

### Participating practices

**Figure 1:**
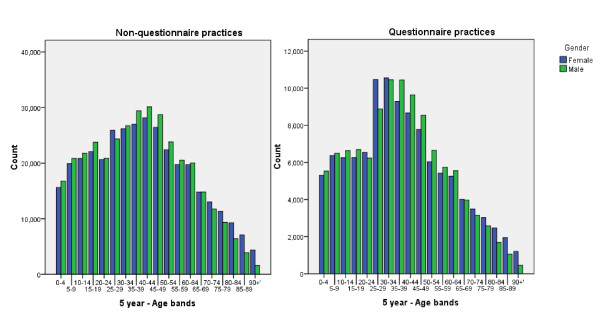
**Age-sex profile of non-questionnaire practices (left) with questionnaire practices (right)**.

We randomly allocated 10 from each study arm as confidence questionnaire evaluation practices (nested study practices). Although randomly allocated the practices in this nested study differ in age profile from the rest of the QICKD trial and the UK national population. The trial population is very close to the national population in terms of its age-sex distribution [21]. The nested study practices represented 14.9% of the study sample population (138,774/930,997) and their practice populations were 1.5 years younger than those in the non-questionnaire practices (mean age 37.0 years, SEM 0.046, compared with 38.5 SEM 0.27; t-test p < 0.001). There was no significant difference in male: female ratio between questionnaire and non-questionnaire practices (Pearson Chi square p = 0.660) (Figure 1). The crude prevalence of CKD in these practices was 5.4%. The age-standardised prevalence of CKD was higher than that of non-questionnaire practices (6.3% vs. 5.3%, OR 1.19: 95% CI 1.19-1.22, p < 0.01).

### Distribution of the questionnaires and reminders

**Figure 2:**
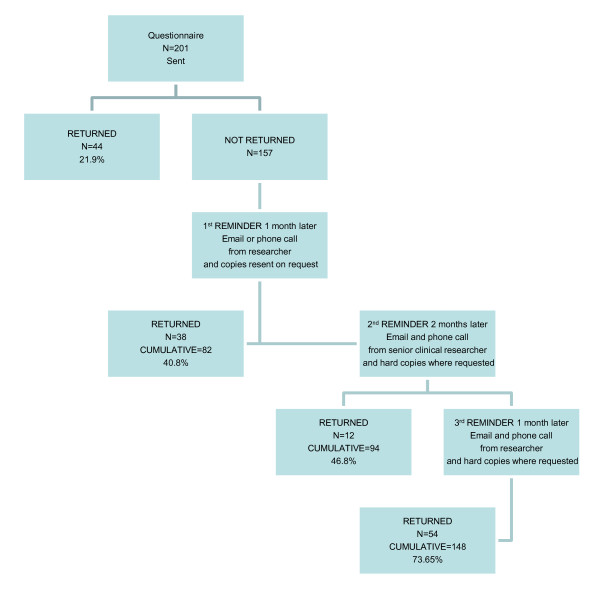
**The scheme of distribution and collection of questionnaires**.

We sent a survey questionnaire to 30 practices (201 individual clinicians in total) and collected responses in the three rounds (Figure 2). After the initial postal distribution three rounds of reminders were conducted. Each round involved a telephone reminder to the participant by a researcher to prompt completion by posting a hard copy or to allow the participant to email a scanned copy to a central administrator. Email reminders were also sent at the same time as the phone calls. The telephone reminder was carried out in as unobtrusive a way as possible.

### Response rate

We sent a survey questionnaire to 30 practices (201 individual clinicians in total) and collected responses in the three rounds (Figure 2). At the end 148 questionnaires were returned (74% response rate) from 93% (28/30) practices.

### Developing a confidence questionnaire

The questionnaire development was carried out in four stages, using an established method [22]. We developed objectives based on our study using knowledge gained from our systematic review [13] and diagnostic analysis [17]. We elected to make comparisons between CKD and other conditions commonly managed in primary care. The objectives of our questionnaire were:

• To compare the confidence of practitioners in controlling systolic BP in patients with CKD with that in patients with hypertension alone. Improved control of systolic BP is the primary outcome measure of the CRT [18].

• To compare confidence in managing proteinuria in CKD with diabetes. Patients with CKD and proteinuria are at high risk of adverse renal and cardiovascular outcomes [10].

• To compare confidence levels in General Practitioner (GP) partners, salaried GPs and nurses. Our systematic review indicated that successful initiatives in CKD had often been non-doctor led [13].

A draft questionnaire was circulated among the investigators who brainstormed the current issues in CKD management. This draft questionnaire was then piloted on practitioners. This cycle was repeated on three occasions. We conducted three focus groups (n = 7, 6, 8) to refine the questionnaire using groups of general practitioners, practice nurses and medical students to validate the questionnaire. These focus groups were facilitated by the principal author to this paper.

The validation process involved the practitioners completing the questionnaire and then marking a colleague's questionnaire while discussing the responses and highlighting potential ambiguities. The focus groups were reviewed and evaluated using a field notebook and transcriptions of tape recordings. A smaller group of GPs were contacted via email (n = 3) to ensure adequate validity.

We elected to include a small number of key competence measures as we felt that we could not interpret confidence entirely isolated from competence. These focussed on knowledge of BP targets for treatment, intervention levels of proteinuria and criteria for referral. The questionnaire consisted of 24 confidence questions rated 1 to 5 (Additional File 1) and 6 knowledge questions. The confidence questions used a Likert scale from 1 to 5, where 1 is *"Not confident at all" *and 5 is *"Very confident."*

### Collecting routine clinical data

We collected anonymised routine clinical data from the computerised medical record systems of the questionnaire practices to enable us to compare confidence with achievement. We extracted the key variables associated with the questionnaire or required to make comparisons: practice identifier, age and gender, BP, significant proteinuria, CKD (strictly applying a standard for chronicity), diabetes (again using strict diagnostic criteria) [23] or hypertension. Data were processed using an established method [24] and analysed in SPSS (Statistical Package for Social Sciences, Version 16).

### Analysis plan

Our analysis had four stages:

1. Comparing confidence levels and knowledge for different age, gender and type of practitioner, and comparing confidence in management of CKD with that of hypertension and diabetes. We judge a person who scored 4 or 5 on the questionnaire to be "confident" and one scoring 1, 2 or 3 to be "not-confident".

2. Establishing BP control and proportion at target for people with CKD, with and without significant proteinuria, diabetes and hypertension.

3. A comparison of level of confidence and achievement of the blood pressure targets set out in national evidence-based guidance [10] and P4P indicators [11]. To do this we calculated a mean confidence score based on the responses from each practice and compared this with the achievement of P4P indicators by that practice.

4. Investigating variation in practice response

### Statistical analysis

We used a range of descriptive and inferential statistical procedures to compare differences in the samples and outcomes. Where appropriate, we report the percentages and 95% confidence intervals (CI) to allow comparison between subgroups. For categorical variables, we used the Pearson Chi square test to detect differences between sub-groups or associations between variables. We performed this analysis in SPSS version 16.

### Ethical Considerations

The study has been approved by the Oxford Research Ethics committee and is a registered clinical trial (ISRCTN56023731).

## Results

The final response rate was 74%. After three rounds of reminders we received 148 questionnaires from 201 primary care professionals from 93% (28/30) of the practices.

**Figure 3:**
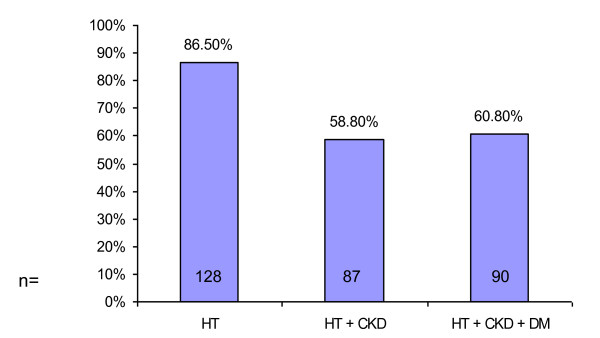
**Confidence in managing hypertension**.

We found that most participants, 86.5% (n = 128; 95% CI: 80.1% to 91.1%) in our study are confident in managing hypertension whereas only 58.8% (n = 87; CI: 50.7% to 66.4%) are confident in the management of hypertension in patients with CKD, and 60.8% (n = 90; CI: 52.7% to 68.3%) are confident in managing hypertension in patients with CKD and diabetes (DM). Just under half of the respondents, 49.3% (n = 73; CI: 41.4% to 57.3%) are confident in achieving lowered blood pressure in patients with CKD (Figure 3).

Importantly, confidence is especially low in the management of patients with CKD and significant proteinuria, who are at higher risk of adverse renal and cardiovascular outcomes. 42.6% (n = 63; CI: 34.9% to 50.6%) of respondents lack confidence with identifying significant proteinuria in patients with CKD and only 41.9% (n = 62; CI: 34.2% to 49.9%) are familiar with using urine protein results to manage CKD. Practitioners are more confident in identifying significant proteinuria in patients with DM, 64.9% (n = 96; CI: 56.9% to 72.1%), and more confident in using urine protein results to manage DM, 47.3% (n = 70; CI: 39.4% to 55.3%) than in patients with CKD.

**Figure 4:**
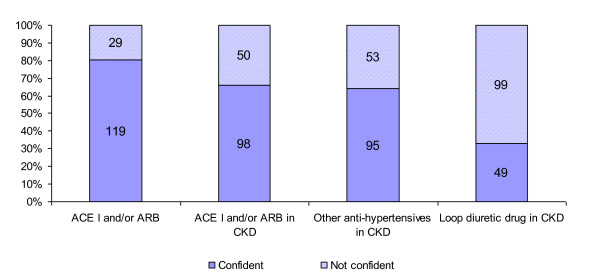
**Confidence in using antihypertensive drugs**.

About a third of respondents lack confidence with using ACE-I and/or ARB in patients with CKD, 33.8% (n = 50; CI: 26.7% to 41.7%). 66.9% (n = 99; CI: 59.0% to 74.0%) of respondents lack confidence with adding a loop diuretic drug (e.g. furosemide) to patients with CKD already on maximum dose of an ACE-I and/or ARB. 35.8% (n = 53; CI: 28.5% to 43.8%) of respondents lack confidence in using anti-hypertensives that are not in the angiotensin modulating category in patients with CKD (Figure 4).

More male GPs reported feeling confident in the overall management of CKD than female GPs, but the difference is not statistically significant (61.5% vs. 50.9%, Pearson Chi Square p = 0.242). Male GPs (salaried GPs and partners) are more confident in managing hypertension in patients with CKD (76.9% vs. 54.5%, p = 0.01) and in managing hypertension in patients with CKD and DM (73.8% vs. 56.4%, p < 0.05) than female GPs. Also male GPs are more confident with using an ACE-I and/or ARB or other anti-hypertensive(s) in patients with CKD (86.2% and 84.6% vs. 65.5% and 63.6%, p < 0.01) compared with their female counterparts.

When it comes to adding a loop diuretic drug (e.g. furosemide) to patients with CKD already on maximum dose of an ACE I and/or ARB, male GPs are approximately twice as confident as female GPs (50.8% vs. 25.5%, p = 0.005).

Confidence in the management of patients with CKD was ranked by respondents' age (Table 1). Younger clinicians (< 35 year old) are more confident in managing hypertension than the 35-54 year old group (100.0% vs. 80.6%, p = 0.023). At the same time, more of the younger (< 35 year old) and more of the older (55 and older) practitioners are confident in the management of hypertension in patients with CKD than the 35-54 year old group (69.6%, 77.4% vs. 50.0%, p = 0.014). 73.9% of the < 35 years old group but only 39.4% of 35-54 years olds are confident in achieving lowered blood pressure in patients with CKD (p = 0.004).

**Table 1:** Confidence in managing CKD and DM in different age groups

Confidence	0-34		35-54		55+		p
	
	N	%	N	%	N	%	
Managing hypertension	23	100.0%	75	80.6%	29	93.5%	0.02
Managing hypertension in patients with CKD	16	69.6%	47	50.0%	24	77.4%	0.01
Managing hypertension in patients with CKD with DM	15	65.2%	52	55.3%	23	74.2%	0.16
Achieving lowered blood pressure in patients with CKD	17	73.9%	37	39.4%	19	61.3%	0.00
Interpreting eGFR to stage CKD	20	87.0%	58	61.7%	25	80.6%	0.02
Monitoring eGFR in patients with CKD	20	87.0%	58	61.7%	24	77.4%	0.03
Monitoring eGFR in CKD patient with DM	16	69.6%	48	51.1%	25	80.6%	0.01
Using urine protein results to manage DM	15	65.2%	37	39.4%	18	58.1%	0.03
Using urine protein results to manage CKD	12	52.2%	35	37.2%	15	48.4%	0.31
Using ACE inhibitors and/or ARB	22	95.7%	71	75.5%	26	83.9%	0.08
Using ACE inhibitors and/or ARB in patients with CKD	19	82.6%	55	58.5%	24	77.4%	0.03
Using other anti-hypertensives in patients with CKD	18	78.3%	54	57.4%	23	74.2%	0.08
Adding a loop diuretic drug (e.g. furosemide) to patients with CKD already on maximum dose of an ACE I and/or ARB	7	30.4%	26	27.7%	16	51.6%	0.05
Using referral guidelines to refer appropriate patients with DM to secondary care	12	52.2%	51	54.3%	26	83.9%	0.01
Using referral guidelines to refer appropriate patients with CKD to secondary care	13	56.5%	43	45.7%	25	80.6%	0.00
Overall management of patients with DM	15	65.2%	54	57.4%	26	83.9%	0.03
Overall management of patients with CKD	9	39.1%	44	46.8%	22	71.0%	0.03

The younger group are significantly more confident than the 35-54 year old group in interpreting eGFR to stage CKD (87% vs. 61.7%, p = 0.02) and monitoring eGFR in patients with CKD (87% vs. 61.7%, p = 0.033), and in monitoring eGFR in CKD patient with DM (69.6% vs. 51.1%, p = 0.01). Only about half of respondents in each group felt confident in using urine protein results to manage DM (47%, p = 0.034) and in CKD (41.9%, p = 0.3).

The > 54 year old group have the highest confidence, compared to the 35-54 year old and < 35 year old groups, in using referral guidelines to refer appropriate patients with either both DM and CKD (83.9% vs. 52.2% vs. 54.3% p = 0.01), or CKD alone (80.6% vs. 56.5% vs. 45.7% p = 0.03) to secondary care.

17.4% and 41.5% of respondents in the < 35 year old group and the 35-54 year old group respectively are not confident with using ACE-I and/or ARB in patients with CKD. This difference was statistically significant (p = 0.03).

86.5% (n = 128) of practitioners were able to correctly identify the P4P thresholds for blood pressure control in patients with CKD without proteinuria compared with 53.4% (n = 79) of respondents when proteinuria is factored in.

Next we evaluated clinical outcomes in the management of blood pressure. The mean systolic BP in the study population as a whole was 125 mmHg and in the population without any cardiovascular (CVS) co-morbidity was 122 mmHg.

Analysis of overall practice performance in BP management revealed poor BP control in patients with CKD and low numbers of patients reaching BP goals (both NICE targets and P4P payment thresholds, Table 2). Only half of patients (53.3%) with CKD and hypertension have achieved the NICE target for blood pressure management whereas this number was 62.5% for people with hypertension without CKD. In patients with CKD and diabetes this figure drops to 29.6%. Currently P4P thresholds for BP are higher than NICE. 67.80% of patients with CKD and DM meet P4P thresholds for BP management.

**Table 2:** Confidence and achievements in BP management in patients with CKD and DM

	Confidence %	BP Mean	Patients at NICE target		Patients at P4P target	
Managing HT	86.5%	138.07	8701	62.5%	11853	85.2%
Managing HT in patients with CKD	58.8%	137.43	2575	53.3%	3141	65.1%
Managing HT in CKD with DM	60.8%	136.26	420	29.6%	963	67.8%
Urine protein results to manage DM	47.3%	135.38	193	32.5%	467	78.6%
Urine protein results to manage CKD	41.9%	140.96	5	21.7%	16	69.6%
ACE-I and/or ARB in HT only	80.4%	138.6	5330	61.4%	7310	84.2%
ACE-I and/or ARB in CKD	66.2%	136.3	2796	48.8%	3841	67.0%
Assessing CVD risk scores in DM	71.6%	128.8	456	71.0%	516	80.4%
Assessing CVD risk scores in CKD	63.5%	129.7	232	73.9%	277	88.2%
Overall management of DM	64.2%	133.2	3556	46.4%	6377	83.2%
Overall management of CKD	50.7%	133.9	6262	56.4%	7932	71.4%

Patients with CKD and both proteinuria and diabetes are a high risk group in whom lowering systolic BP is particularly important. There appears to be a lack of confidence in treating these patients and this is reinforced by a low achievement of BP targets, with only 31.3% patients meeting at goal of 130 mmHg as recommended by NICE.

70% of patients with HT received angiotensin modulating drugs for blood pressure treatment (mean BP achieved 138.6 mmHg). 61.4% of these patients meet NICE targets and 84.2% meet P4P thresholds for BP control.

79% of CKD patients (mean 136.3) received ACE-I or ARB. However, in the same group, only 48.8% (NICE) and 67.0% (P4P) of patients are at target for BP control. Practitioner confidence is also low so far as treatment of these conditions are concerned.

Practitioner confidence is lowest in managing the following groups; patient with CKD, with DM and proteinuria, and with CKD and proteinuria (58.1%, 47.3% and 41.9% respectively). CKD combined with DM or HT or both produce higher confidence values than that of CKD alone.

**Figure 5:**
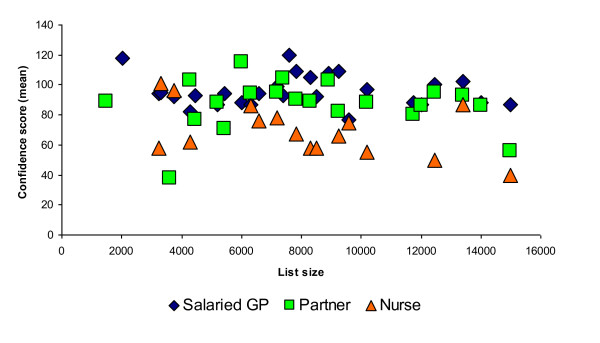
**Salaried GP, GP Partner and Practice Nurse scores across practice list size**.

We compared individual practice responses to see if outliers or practice variation might account for a difference in confidence levels. We found no difference related to list size and similar results for the different patient groups (Figure 5).

## Discussion

### Principal findings

Practitioners are less confident in managing CKD than hypertension or diabetes. There is also a lack of confidence in managing proteinuria, combined with a knowledge gap in interpreting proteinuria test results.

Within the respondent group, salaried doctors, males, older and younger doctors and GPs compared to nurses were more confident in managing CKD. Clinicians are least confident in managing individuals with CKD who are at highest risk, i.e. those with proteinuria. Confidence is lower in managing patients with proteinuria and CKD than those patients with proteinuria and diabetes.

The quality of care in CKD, measured by ascertainment of standards in national guidance and for P4P is lower where confidence is low. This is particularly apparent in CKD and in people with CKD and diabetes.

Nearly all (> 80%) of people with hypertension taking an angiotensin modulating drug achieve national quality targets compared with two-thirds of people with CKD.

### Implications for practice

Low practitioner confidence and knowledge gaps may be reversed by educational interventions. The QICKD study, using information (guidelines and prompts) and education (audit based education) will inform whether these interventions change confidence and ultimately the quality of care. These interventions were chosen as they represented two common initiatives used in primary care to improve care and thought to prompt change in practice.

Interventions may need to be tailored to meet the needs of different practitioner groups, as there are marked differences between the groups of practitioners. Nurses in particular should have specific training as this clinician group deliver much of the chronic disease management in primary care and increasingly chronic kidney disease management. One consequence of P4P may be that practices improve quality up to the standard required to achieve the indicator. The highest risk patients, who may also be the most difficult to manage, may not be optimally treated. Incentives could be tailored to reward achievement of NICE thresholds for the highest risk patients, for example the use of Local Enhanced Services in Primary Care Trusts in England.

### Comparison with the literature

Other studies have shown a knowledge gap in CKD management [25,26] reinforcing the findings of our own diagnostic analysis [17]. There are some pointers from educational research and from other disease areas that level of knowledge [27,28] and confidence is associated with improved practice [29-32]; however most of these articles are descriptive rather than conducted in trials.

It may be premature to presume that the patterns of practitioner confidence described in our study are generaliseable. One study of knowledge of CKD showed it declined with increasing practitioner age [25]. Another, in the field of dementia [31] suggested younger and female GPs had greater knowledge and awareness; whereas the exploration of discussing sexual difficulties showed no gender difference [32].

### Limitation of the method

There may be a number of sources of bias in this investigation. Firstly, the practices participating in the QICKD study are volunteer practices who may not be representative of the wider population. Secondly, we have no information about the non-responders.

We can only look at comparing individual practitioners' responses with practice level outcome data. This is because most practices allow patients to see a number of doctors.

### Call for future research

We need to determine the cost effectiveness of any educational intervention updating economic modelling based on previous guidance [33]. Further studies which provide educational or other interventions to people who lack confidence in managing a condition should be conducted to determine whether this improves the quality of care.

## Conclusion

Practitioners are less confident in managing CKD than hypertension or diabetes and are less likely to achieve BP targets where confidence is low. Improving knowledge and confidence may provide the key to improving the quality of CKD management in primary care, especially for higher risk patients with diabetes and with proteinuria.

## Competing interests

AT^1,2 ^Nil declared

OD^2 ^Nil declared

SdeL^2 ^SdeL is the GP expert advisor for the Quality and Outcomes Framework (QOF - a pay-for-performance (P4P) scheme) with the role of developing a CKD Indicator. This was done on behalf of NICE (National Institute for Health and Clinical Excellence). SdeL has received funding for research staff from Roche for the data analysis which formed part of the NEOERICA study. He has received sponsorship from Pfizer to speak at two cardiovascular meetings in 2008; received an honorarium for writing a magazine article (Prescriber) jointly with HG.

JvV^2 ^For two years JvV's salary was part-funded by the NEOERICA study

TC^2 ^Nil declared

RG^1 ^Nil declared

KH^3 ^Funding: Grants Pfizer International Doxazosin Award 2003: The role of alpha blockade on matrix synthesis by mesangial cells - £10,000; Pfizer award 2004: To investigate the effect of atorvastatin on renal reperfusion injury - £12,000; Health Foundation 2007-2010: Quality Improvement in CKD: a challenge for primary care - £695,000; Edith Murphy Foundation 2007-2010: Quality Improvement in CKD due to diabetes - £450,000; LNR CLAHRC 2008-2014: Prevention of Chronic Disease and its Associated Co-Morbidity theme - c£4 million out of c£20 million total. Funding: others in last 5 years (travel support & ad hoc honararia) Roche, Ortho Biotech, Amgen, Baxter, Boehringer. Other: Advisory Board Membership Roche, Genzyme, Shire, Baxter, Novartis.

CT^4 ^Dr Tomson was a member of advisory board of Genzyme UK between April 2002 and May2004. He had received honoraria from Janssen-Cilag, MSD, Bayer, Novartis, Baxter Healthcare, Amgen, Genzyme, AstraZeneca, Pfizer, Roche. He is a current member of International Steering Committee of the Study of Heart and Renal Protection.

NT^5 ^Funding: Grants Hospital Savings Association - £5,000; Kidney Research UK/British Renal Society - £45,000; Insulin Dependent Diabetes Trust - £7,000; SW Thames Kidney Fund - £10,000. Funding: others in last 5 years: Baxter Healthcare, Roche, Novartis.

HG^1,6 ^Hugh Gallagher is an Honorary Senior Lecturer at St. George's and a renal physician at St. Helier Hospital with clinical responsibility for renal medicine in southwest Surrey. He is a panel member expert advisor for the QOF. He has received funding from several pharmaceutical companies for educational presentations on CKD, and an honorarium from a GP magazine to write an article on CKD (jointly with SdeL).

## Authors' contributions

AT^1,2 ^: Developed and validated confidence questionnaire, contributed to analysis of data, contributed to each version of the paper. OD^2 ^: Conducted major role in analysis of data, created 1^st ^draft and contributed to each version. SdeL^2 ^: Developed the idea of linking confidence to live practice data, contributed to analysis of data, major part in drafting and redrafting paper, PI of QICKD study. JvV^2 ^: Cleaned and analysed raw practice data, contributed to final version of paper. TC^2^: Contributing to developing questionnaire, analysis of data, contributed to final version of paper. RG^1 ^: Major Input to early analysis of data, contributed to final version of paper. KH^3^: Contributed to Research Idea, contributed to final version of paper. CT^4^: Contributed to Research Idea, contributed to final version of paper. NT^5 ^: Contributed to Research Idea, contributed to final version of paper. HG^1,6 ^: Contributed to Research Idea, contributed to final version of paper.

All authors declare that the answers to the questions on your competing interest form are all "No". All authors have read and approved the final manuscript.

## Pre-publication history

The pre-publication history for this paper can be accessed here:

http://www.biomedcentral.com/1471-2296/12/83/prepub

## Supplementary Material

Additional file 1 **Questionnaire: How confident are General Practitioners & the Primary care team in managing Chronic Kidney Disease (CKD)**. Click here for file
